# Facing the CDK4/6i resistance dilemma in patients with breast cancer, exploration of the resistance mechanism and possible reverse strategy: A narrative review

**DOI:** 10.1097/MD.0000000000032238

**Published:** 2022-12-23

**Authors:** Jiayi Wu, Wei Wang, Xiying Shao, Guang Lin, Xiaojia Wang

**Affiliations:** a Department of Clinical Medicine, Zhejiang Chinese Medical University, Hangzhou, China; b Department of Clinical Medicine, Wenzhou Medical University, Wenzhou, China; c Department of Breast Medicine, Zhejiang Cancer Hospital, Hangzhou, China.

**Keywords:** biomarker, reverse treatment strategies, breast cancer, CDK4/6i, resistance mechanism

## Abstract

Breast cancer is one of the highest rates of malignancy of women, approximate 70% metastatic breast cancer are hormone receptor positive (HR+) and human epidermal growth factor receptor 2 negative (HER2−). Hormone therapy is the primary strategy of HR+/HER2− metastatic breast cancer. With the permission of cyclin-dependent kinase 4 and 6 inhibitors (CDK4/6i), progress free survival and overall survival were significantly licensed. However, inevitable outcome of CDK4/6i resistance has become the main reason that restricts the clinical benefit of patients. In recent years, the research on dealing with drug resistance has become a hot topic, a large number of molecular mechanisms have been focused, and a lot of experiments have been carried out at the preclinical level. This review summarizes the current knowledge of CDK4/6i resistance mechanism, systematically expounds the signaling pathways and targets leading to CDK4/6i resistance, analyzes different ways and mechanisms, and provides theoretical guidance for the clinical reversal of endocrine therapy resistance.

## 1. Introduction

Breast cancer has become the world-health issue, the first morbidity and second mortality of women.^[1]^ Although the 5-year overall survival (OS) has reached to 90%, the 5-year OS was only 25% in metastatic or advanced breast cancer (MBC/ABC).^[2]^

Hormone receptor positive (HR+)/human epidermal growth factor receptor 2 negative (HER2−) BC accounts for approximately two-thirds of all breast cancers and as a result, hormone therapy plays an integral role in breast cancer patients. A mass of clinical trials and meta-analyses have confirmed that there is no significant difference in clinical benefits between endocrine therapy and traditional chemotherapy, while the adverse reactions of endocrine therapy are far less than that of traditional chemotherapy.^[3]^ However, primary or acquired resistance has inevitable developed. 30% HR + MBC patients show primary resistance and most of them initially sensitive to hormonal therapy ultimately display resistance. The most common mechanisms include activation of signaling pathways and alteration of cell cycle pathways. Cyclin-dependent kinase 4 and 6 inhibitors (CDK4/6i) is a small molecule drug, taken orally, that dephosphorylates the retinoblastoma (Rb) protein by targeting CDK4 and 6.^[4]^ The Rb protein dephosphorylation results in a cell cycle arrest blocking the progression of tumor cells from G1 to S phase.

**Figure 1.**
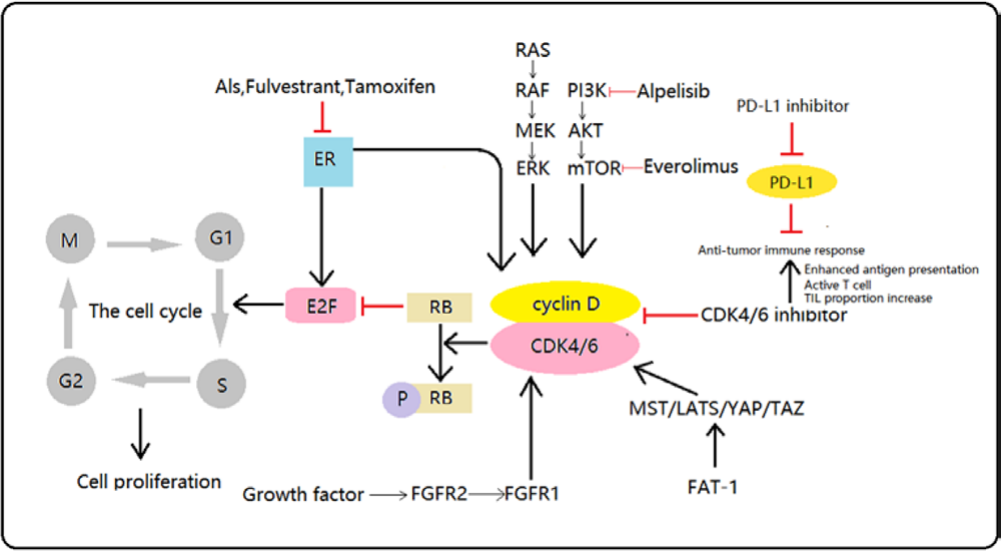
This model explains the mechanism by which CDK4/6i blocks G1/S changes in the cell cycle. The model describes the checkpoint, regulatory protein, upstream and downstream pathways (PI3K/Akt/mTOR, FGFR, Ras/Raf/MEK and more) that may lead to CDK4/6i resistance. Immunization will be discussed in a later section. CDK4/6i = cyclin-dependent kinase 4 and 6 inhibitors, FGFR = fibroblast growth factor receptor.

In fact, the identification of cyclins and CDKs was reported as early as the 1970s, and this discovery was recognized by the Nobel in 2001.^[5]^ CDK4/6i targets the CDK-RB1-E2F pathway, which is vital in the progression of the cell cycle and does not function properly in most cancers.^[6]^ In breast cancer (BC), the activation of estrogen receptors (ER) triggers the complexation of CDK4/6 with cyclin D1. The interaction of CDK4/6 to cyclin D1 leads to phosphorylation of the Rb protein, providing the initiating signal for tumor cell proliferation. In addition, CDK4/6’s binding partner cyclin D1 is frequently overexpressed in HR+/HER2− BC patients, resulting in sustained activation of the Cyclin D1-CDK4/6 + complex. CDK4/6i can result in complete dephosphorylation of Rb, leading to inhibition of cell cycle progression by leading to non-release of the transcription factor E2F.^[7]^ Figure 1 exhibited CDK4/6 inhibitors in cancer cells.

In addition to the classical mechanism of cell cycle, the latest preclinical studies have uncovered additional mechanisms by which CDK4/6i can treat tumor progression in vivo.^[8]^ CDK4/6i can induce cell senescence after cell cycle arrest and prevent cells from entering the cell cycle.^[9]^ CDK4/6i can also change cell metabolism and induce cell apoptosis.^[8,10]^ Moreover, plentiful studies have confirmed the immunoregulatory role of CDK4/6i, which synergistic immune mechanism inhibits the proliferation of tumor cells by enhancing the antigenicity of tumor cells, previous preclinical studies have shown that CDK4/6i is more sensitive to luminal subtype of human BC cell lines than other subtypes.^[8]^

### 1.1. Clinical application of CDK4/6is

As the first CDK4/6i approved through fast-track approval process by the FDA in 2015, palbociclib was initially in first-line endocrinotherapy for the treatment of post-menopausal women with HR+/HER2− ABC limited in combined therapy with letrozole.^[11]^ But soon, the combination of palbociclib with any aromatase inhibitor was also approved on March 31, 2017, and subsequently, in February 2016, palbociclib was approved by the FDA for use in HR+/HER2− ABC patients who progressed after endocrine therapy. On September 16, 2016, the European Medicines Agency also made the first official approval of a CDK4/6 inhibitor.^[12]^ All of these approvals are supported by evidence from the PALOMA series of clinical trials (one phase II and three phase III).^[4]^

Ribociclib is another highly selective, reversible CDK4/6i. As a new class of targeted small molecule drugs, ribociclib can selectively block Rb protein phosphorylation process to block tumor proliferation, and thus play an anti-tumor role.^[13,14]^ The MONALEESA series of clinical studies on Ribociclib included MONALEESA-2, MONALEESA-3 and MONALEESA-7.

As the only CDK4/6i approved as monotherapy for the treatment of ER+/HER2− ABC or MBC, abemaciclib (LY2835219) has entered Medicare in January 2022, and is an orally available CDK4/6i that seems to differ from palbociclib and ribociclib as it has a possible inhibition of CDK9.^[6]^ And it is the most potent of the three CDK4/6is.^[11,15]^ Table 1 summarizes the clinical research evidence supporting the inclusion of CDK4/6i in clinical guidelines. These data include 3 series of clinical studies related to 3 inhibitors, including disease-free survival, OS, objective response rate and other important indicators.

**Table 1:** Historical clinical evidence supporting the clinical use of three CDK4/6i therapies in HR+/HER2− patients (does not need color in print). CDK4/6i = cyclin-dependent kinase 4 and 6 inhibitors.

CDKi	Registration trial	Lines	Phase	Patient number	Menstrual status	Treatment	PFS (mo)	OS (mo)	ORR (%)
palbociclib	PALOMA-1(NCT00721409)	1^st^	II	165	Post	Letrozole + palbociclib/Letrozole	10.2 vs 20.2	33.3 vs 37.8	33 vs 43
	PALOMA-2(NCT01740427)	1^st^	III	666	Post/Pre	Letrozole + palbociclib/Letrozole	14.5 vs 24.8	46.8 vs 51.8	35 vs 42
	PALOMA-3(NCT01942135)	≥1^st^	III	521	Post/Pre	Fulvestrant ± palbociclib	4.6 vs 9.5	28.0 vs 35.0	11.1 vs 25
	PALOMA-4(NCT02297438)	1^st^	III	340	Post	Letrozole + palbociclib/Letrozole	13.9 vs 21.5	NA	38 vs 43.4
ribociclib	MONALEESA-2(NCT01958021)	1^st^	III	668	Post	Letrozole ± ribociclib	16 vs 25.3	51.4 vs 63.9	37 vs 53
	MONALEESA-3(NCT02422615)	≥1^st^	III	726	Post	Fulvestrant ± ribociclib	12.8 vs 20.5	33.7 vs 39.7	29 vs 41
	MONALEESA-7(NCT02278120)	1^st^	III	672	Pre	NSAI/Tamoxifen + OFS ± ribociclib	13.0 vs 23.8	48 vs 58.7	36 vs 51
Abemaciclib	MONARCH-1(NCT02102490)	≥1^st^	II	132	NA	Abemaciclib monotherapy	6.0	17.7	20
	MONARCH-2(NCT02107703)	≥1^st^	III	669	Post	Fulvestrant ± abemaciclib	9.3 vs 16.4	37.3 vs 46.7	21 vs 48
	MONARCH-3(NCT02246621)	1^st^	III	493	Post/Pre	NSAI ± abemaciclib	14.8 vs 28.2	54.5 vs 67.1	44 vs 59
Dalpiciclib	DAWNA-1 (NCT03927456 )	≥1^st^	III	357	Post/Pre	Fulvestrant ± dalpiciclib	7.2 vs 16.6	NA	23.3 vs 35.7
	DAWNA-2(NCT03966898)	1^st^	III	456	Post/Pre	Anastrozole/Letrozole ± dalpiciclib	18.2 vs 30.6	NA	57.4 vs 62.4

Menstrual status = menstrual status of patients at the time of enrollment into the clinical study, NA = not available, NSAI = nonsteroidal aromatase inhibitors, ORR = objective response rate, OS = overall survival, PFS = progression free survival.

## 2. About the CDK4/6i drug resistance mechanism

### 2.1. Cell cycle specific CDK4/6i resistance mechanism

Tumor suppressor Rb, an indispensable checkpoint in the classical cell cycle, is considered to be a target of many endocrine drugs and a marker of sensitivity. It comes in 2 states, phosphorylated and dephosphorylated.^[3]^ The Rb gene, as one of the oldest studied tumor suppressor genes, is critical for cancer suppression, regulating cell growth and controlling cell division to some extent. Rb prevents other proteins from triggering DNA replication, a process by which DNA replicates itself. And Rb’s strict regulation of DNA replication prevents normal cells from turning to malignant proliferation.

Since the complete Rb pathway is essential for CDK4/6i, Rb pathway proteins has been proven to be as in vitro biomarkers.^[3,16]^ For example, a recent study showed that complete G1/S transmission is a reliable prognostic marker for HR+/HER2− BC patients.^[16]^ Finally, the study of Abemaciclib shows that the expression level of ER and Rb can influence the curative effect to Abemaciclib.^[17]^ Another study found that tumors with RB1 mutations or deletions were highly sensitive to Aurora kinase inhibitors, suggesting the possibility of reversing CDK4/6i resistance.^[18]^

Cyclin Dependent Kinase Inhibitor 2A, encoding p16INK4A, results in uncontrolled activation of CDK4/6.^[8]^ Dysregulation of INK4 is associated with ET resistance.^[19]^ p16INK4A has been confirmed by multiple studies as a frequent target of inactivated mutations and deletions in cancer.^[20]^ Tumors with high P16INK4A content are resistant to CDK4/6i.^[21]^ In HR+/HER2− BC, the loss of Rb was minimal, while cyclin D1 was overexpressed or amplified in the majority of cases in all cancer patients.^[22]^ Another study has demonstrated that the presence of Rb and the loss of p16 determine the effect of palbociclib.^[23,24]^

CDK4 is a key target of CDK4/6i blocking the cyclin pathway.^[19]^ Amplification and overexpression of CDK4 are associated with reduced sensitivity to CDKi, limiting the efficacy of CDK4/6i. Studies of alveolar rhabdomyosarcoma cell lines and xenograft models RH28 and RH41 have shown reduced activity of CDK4/6i in cells that overexpress CDK4. And CDK4-overexpressing glioma cells were found to be absolutely resistant to CDK4/6i.^[25]^ In the PALOMA-2 study, CDK4 and CDK6 expression was not significantly associated with pabociclib and letrozole treatment.

CDK6 plays a pivotal role in cell cycle progression.^[19]^ FAT1 loss induces CDK6 expression through the Hippo pathway and plays an important role in CDK6 amplification, and this is the hallmark of CDK4/6i resistance.^[26]^ In a clinical study, targeted sequencing was performed on 348 tumor samples obtained before patients were treated with CDK4/6i, and the results showed that loss of functional mutations in the FAT1 tumor suppressor gene leads to resistance to CDK4/6i, and FAT1 upregulates CDK6 expression through the HIPPO pathway.^[27]^

Overexpression of CCNE1 encoding cycline is a widely accepted mechanism of resistance to CDK4/6i.^[28,29]^ CDK4/6i resistant cells bypass the classical signaling pathway and instead use a bypass signaling pathway via CDK2.^[30]^ Another preclinical study showed that CCNE1 was related to adverse reactions to Palbociclib in tumor specimens.^[23]^ Combination therapy with CDK2 and CDK4/6 inhibition overcame Palbociclib resistance in an anti-palbociclib transplant model in a cell experiment.^[31]^ CDK4/6i resistance mediated by CCNE-amplification bypasses the cyclinD1-CDK4/6 signaling pathway and instead passes through the CCNE-CDK2 signaling pathway, which has been strongly demonstrated. In drug-resistant patients with high CCNE, CDK2 expression, target blocking of the CCNE-CDK2 bypass pathway and its upstream targets may have potential research value.

The loss of Rb is related to the overexpression of E2F.^[19]^ Different studies have shown that E2F inhibits miR-223 transcription by combining with its promoter, suggesting that E2F1 can be used as miR-223 gene, and inhibition of miR-223 is a common feature in the process of cancer progression.^[32]^ E2F amplification allowed it to bypass CDK4/6i and block the cyclin pathway, leading to cancer progression after CDK4/6i treatment. This suggests that CDK4/6i combined with targeted therapies in the downstream E2F pathway may overcome the clinical benefit of drug resistance in E2F-amplified resistant patients.

MDM2 gene is an important oncogene. The P90 protein encoded by MDM2 is a protein that negatively regulates the activity of p53, which can bind to p53 and make the p53 gene lose its normal function, and has the effects of destroying cell stability and inhibiting cell aging. MDM2 overexpression accounted for 20% of BC patients, which is particularly helpful in promoting the progression of HR-positive BC.^[19]^ CDK4/6i resistant cells have been reported to disrupt the aging pathway and to be insensitive to induction of aging. Thus, MDM2 interrupting the aging pathway in a p53-dependent manner may interfere with CDK inhibitors blocking cancer cell proliferation through the aging pathway.^[19]^ Next, a study examined whether biomarkers of CDK4/6i and MDM2i responses could be used in vitrand down-regulation of the Ki67 index occurred when these drugs were used in vivo, while resistant tumors in vivo had a higher baseline Ki67 index but were not powerfully impacted by in vivo treatment.^[33]^ Therapeutic resistance to CDK4/6i was neutralized with MDM2 inhibitors, which induce p21 by stabilizing p53.^[34]^ The combination of another MDM2 inhibitor (CGM097) with a CDK4/6i showed a synergistic effect in eliminating cells resistant to CDK4/6is.^[19]^

Poly ADP-Ribose Polymerase (PARP) can repair DNA and cleave the core member of cell apoptosis, caspase. DNA damage leads to DNA mutations and is important in the uncontrolled proliferation of tumor cells.^[35–37]^ Targeting DNA damage responses in cancer by inhibiting PARP provides an important therapeutic strategy. Importantly, PARP’s small-molecule compound inhibitors disrupt the escape pathways of tumor cells and sustain DNA damage, leading to stagnation of tumor cells or cell death.^[37,38]^ Since DNA repair is inhibited in G1/S phase cells, there are theoretical evidences for the efficacy of this combination therapy.^[39]^ In conclusion, PARPis showed an exciting reversal of resistance in Rb-lost CDK4/6i resistant BC cells, the U.S. FDA has approved PARPis to treat ovarian cancer, but despite this, the molecular mechanism of how PARPis can achieve this reversal is still not clear.

### 2.2. Non-cell cycle specific CDK4/6i resistance mechanisms

Studies have shown that E2F1 can act as a transcriptional suppressor of miR-223 gene.^[40,41]^ In addition, a recent study found that down-regulation of miR-223 is an early step in the development of BC. Treatment with CDK4/6i inhibited E2F and restored miR-223 expression. It is suggested that miR-223 may be a predictive biomarker for CDK4/6i response, and its loss may identify lesions that may develope to invasive BC. In this study, miR-223 overexpression decreased the carcinogenic potential of HER2-driven transformed breast epithelial cells.^[42]^ In one experiment, increased miR-223 expression can be detected in the palbociclib resistance model.^[34]^ In conclusion, the collected data strongly point to miR-223 as an active factor in CDK4/6i resistance.

Fibroblast growth factor receptor (FGFR) signaling pathway is activated in a variety of tumor types including BC.^[43,44]^ Among the five FGFRs, FGFR1-4 has been confirmed that they cancer progression, CDK4/6 resistance, and endocrine resistance.^[45]^ Studies have shown that FGFR1 amplification activates PI3K/Akt and Ras/MEK/ERK signaling pathways in endocrine resistant BC cells.^[46]^ The FGFR pathway is activated primarily by FGFR2 amplification, which allows FGFR1 to mediate endocrine resistance. And mutation of FGFR1 is directly related to palbociclib resistance.^[47]^

PI3K-Akt- mTOR is one of the three important signaling pathways that have been identified as cancer, and is also one of the most thoroughly studied among many signaling pathways.^[19]^ Activation of PI3K/Akt/mTOR signaling pathway is abundant in hormone-dependent BC. In addition, the association between the PIK3/Akt/mTOR pathway and CDK4/6i resistance has also been frequently reported. CDK4/6i resistant BC cells evade the control of ER signaling through PI3K/Akt/mTOR signaling pathway and CDK4/6i activates the PI3K/Akt pathway by PDK1 phosphorylation of Akt at S477/T479. Notably, S477/T479 is a CDK2-dependent phosphorylation site.^[31]^ PDXs in CDK4/6i resistant melanoma often shows activation of the PI3K -Akt pathway, inhibiting this pathway to improve CDK4/6i response in a p21-dependent manner.^[19]^ A melanoma model showed that the PI3K/Akt pathway has been shown to mediate CDK4/6i resistance by inhibiting P21.^[48]^

Ras gene activation constitutes oncogenes, which are evolutionarily highly conserved and widely present in a variety of eukaryotic cells. H-ras, K-Ras and N-RAS all belong to the Mammalian Ras protein family. Ras is a GTP/GDP regulating the transmission of signal pathway through the mutual transformation of GTP and GDP. Ras protein configuration changes, causing cell proliferation, malignant transformation. Preclinical study on melanoma discovered that dual targeting of CDK4/6 and mutated BRAF or MEK resulted in BRAF and NRAS mutated melanoma.^[30]^ Meanwhile, a study found that up-regulation or mutation of NRAS was related to the CDK4/6i resistance both in vivo model and clinical case, respectively.^[3]^ Mutated BRAF and NRAS melanomas acquired resistance to inhibition of CDK4/6 by upregulating mTOR signaling.^[49]^

### 2.3. Exploration of biomarkers to predict the efficacy and resistance of CDK4/6i

The occurrence and progression of tumors are often caused by the co-alteration of multiple pathways involved in multiple genes, and even different kinds of mutations in the same gene can produce different results.^[50]^ HR positivity and HER2 negativity are the only condition we have for defining the scope of application of CDK4/6i. Attempts to find biomarkers to predict palbociclib from more than a dozen candidates in the Paloma-3 study failed.^[51]^ Similarly, in PALOMA-2, ER and ESR1 levels could not be correlated with the efficacy of CDK inhibitors. In a comprehensive analysis of the MONARCH series of studies, PR levels did not significantly distinguish abemaciclib effects.^[52]^ However, in our real-world study, both Ki67 ≥ 14% or 30% and PR < 20% predict worse clinical outcomes of CDK4/6i and hormone therapy.^[53]^ Moreover, early treatment-associated neutropenia has been shown to predict response to palbociclib.^[54]^ Naturally, most biomarker studies have focused on genes related to the mechanism of action of CDK inhibitors. But in PALOMA-3, a variety of alternative biomarkers failed to correlate with the efficacy of palbociclib.^[55]^ Similar attempts in MONALEESA-2 failed to find a reliable biomarker for ribociclib.^[56]^ And this was confirmed by Neven’s team in a study published at ESMO 2018.^[3]^ As mentioned above, although multiple clinical studies do not support a significantly different effect of any single gene mutation on CDK4/6i efficacy, however A clinical trial sequenced the genes (CDK4/6 amplification, CCND1/2/3 alteration, or Cyclin Dependent Kinase Inhibitor 2A/B alteration) of 40 patients treated with a CDK inhibitor. Studies have found that patients with metastatic cancer have about 2 to 5 deleterious genomic changes. A significant increase in progress free survival (PFS) was observed when the CDK4/6 inhibitor was used in combination with other drugs that matched these gene co-alteration.^[55]^ This suggests that resistance can also be induced by a combination of mutations in multiple gene mechanisms or by driver feedback loops. Moreover, a mathematical predictive model to develop sequential treatment plans, enables a degree of individualization of treatment and avoidance of resistance.^[57]^ This model constructs a relatively simple logical model to reflect the relationship between different resistance mechanisms.^[58]^ Of course, this study alone is not enough to support the whole idea, but it shows the model’s potential to overcome the problem of drug resistance and prevent tumor proliferation by not depending on any single therapy.

## 3. Treatment strategies for reversing CDK4/6i resistance

Table 2 summarizes clinical studies of more combination therapies with CDK inhibitors and Table 3 collects ongoing clinical studies of post-treatment progress with CDK inhibitors.

**Table 2:** Clinical study of CDK inhibitor combination therapy developed to respond to drug resistance.

Trial	Phase	Number of patients enrolled	Patient population	Study arm	Recruiting status
NCT02088684	Ib/II	70	Patients with ER+/HER2− locally advanced or metastatic breast cancer	Phase Ib: Ribociclib with fulvestrant; Ribociclib and BKM120 (PI3K-pan class I-inhibitor) with fulvestrant; Ribociclib and BYL719 (PI3K-alpha specific class I inhibitor) with fulvestrant.	Completed
NCT02871791	I/II	41	Human Epidermal Growth Factor 2 Negative HR + Breast Cancer	Palbociclib + Everolimus + Exemestane	Completed
NCT01857193	I	132	Patients with ER+/HER2− advanced breast cancer	Ribociclib + everolimus + exemestane	Completed
NCT02057133	Ib	198 (Estimated Enrollment)	Metastatic breast cancer	Abemaciclib in combination therapies (letrozole, anastrozole, tamoxifen, exemestane, exemestane plus everolimus, trastuzumab, LY3023414 plus fulvestrant, pertuzumab plus trastuzumab with loperamide, or ongoing endocrine therapy)	Active, not recruiting
TRINTI-1 NCT02732119	I/II	104	Locally advanced metastatic breast cancer following treatment with a CDK 4/6 inhibitor	Ribociclib + everolimus + exemestane	Completed
NCT02779751	Ib	100 (Estimated Enrollment)	Patients With Stage IV Non-Small Cell Lung Cancer or HR+/HER2− Breast Cancer	Abemaciclib + pembrolizumab	Active, not recruiting

**Table 3:** Ongoing studies in patients with advanced CDK inhibitors.

Registration trial	Phase	Previous CDK4/6i	Treatment
MAINTAIN (NCT02632045)	II	Al + palbociclib/ribociclib	Ribociclib + fulvestrant versus placebo + fulvestrant
NCT02738866	II	Palbociclib + Al	Palbociclib + fulvestrant
EMERALD (NCT03778931)	III	Elacetrant	CDK4/6i + AI or fulvestrant
Bylieve NCT03056755	II	CDKi + Al	alpelisib + fulvestrant or letrozole
PACE (NCT03147284)	II	CDK4/6i + based regiment	Fulvestrant versus palbociclib + fulvestrant versus -avelumab
NCT03238196	I	Palbociclib	Palbociclib + fulvestrant + erdafitinib(FGFRi)
MORPHEUS (NCT03280563)	I/II	CDK4/6i	Fulvestrant/Atezolizumab + entinostat/Atezolizumab + fulvestrant/Atezolizumab + ipatasertib/Atezolizumab + ipatasertib + fulvestrant Atezolizumab_ + bevacizumab + ET/Atezolizumab + abemaciclib + fulvestrant

CDK4/6i = cyclin-dependent kinase 4 and 6 inhibitors, FGFR = fibroblast growth factor receptor.

### 3.1. PI3Ki/ET/CDK4/6i triple therapy

Everolimus, is an mTOR inhibitor. As the first clinically approved targeted drug to reverse endocrine drug resistance, Everolimus has naturally become the highlight of the most attention in reversing CDK4/6i resistance.^[18]^ In recent years, adding Everolimus to ET for patients with ABC has become a trend, including various combinations.^[56]^ Clinically, patients with ER+/HER2− BC treated with Everolimus have been shown to be beneficial, with a significant improvement in PFS in the Everolimus + exemestane group compared to patients previously treated with NSAI.^[57]^

In a preliminary analysis of a Phase 1/2 TRINTI-1 study, it met its primary efficacy endpoint and was the first trial to demonstrate the activity and tolerability of ET + mTORi + CDK4/6i sequential triotherapy.^[58]^ In TRINTI, most of these have progressed with aromatase inhibitors and CDK4/6i. TRINTI-1 showed an acceptable toxicity profile with an initial efficacy of 33% PFS at 1 year. Although the concept that the triple combination can benefit patients with CDKi resistance cannot be proved, this should be further explored.^[59]^ Bylieve is a study of alpelisib plus fulvestrant or letrozole after the progression of CDKi treatment. Although the fulvestrant group appeared to outperform the letrozole group in the first interim analysis, the data are still immature.^[58]^ To date, only alpelisib has been approved by FDA. This is largely because reliable long-term OS results from clinical studies are still scarce.^[18]^

### 3.2. CDK4/6i combined immunotherapy

In recent years, the application of immune checkpoint blockers in BC has become a research hotspot, and many immune-related studies are being carried out. Although most of these studies have focused on triple-negative BC, there have been many positive results from studies of combined immune and endocrine therapy.^[19]^ There is now considerable research evidence demonstrating the efficacy of combined inhibition of CDK and immune checkpoints. CDK4/6 inhibitors enhance the efficacy of PD-L1 by increasing antigen presentation, activating T cells, and increasing the proportion of tumor-infiltrating lymphocytes.^[57]^

### 3.3. Other experimental strategies to reverse resistance

Notably, FGFR inhibitors/CDK4/6i targeting ESR1 mutations, oral SERDs and SERMs, and other pathways driving CDK4/6i resistance are all potent example combinations currently under investigation.^[20]^ Previous studies have shown that DNA repair is highly inhibited during G1/S phase, mediating the progression of Rb-deficient cancer cells through CDK4/6. However, the combination of PARPi and CDK4/6i is not expected to show synergism in Rb-deficient cells either.^[40]^ Combined inhibition of the CDK4/6 and FGFR pathways may prevent tumor cells from bypassing the cell cycle to proliferate through the FGFR-MAPK axis.^[19]^ The study, led by Carlos Arteaga, MD, of UT Southwestern Medical Center, suggests that a combination of FGFR inhibitors and CDK4/6is plus endocrine therapy has a potential role. This study is a phase 1 trial (NCT03238196) evaluating the addition of Balversa to palbociclib and fulvestrant in patients with FGFR-amplified estrogen receptor ER + HER2− BC. The preliminary results of this trial demonstrate the activity of triple therapy in FGFR-amplified populations.^[60,61]^ Finally, a study found that CDK4/6i combined with metformin significantly promoted cell cycle arrest, which related to another mechanism of action other than the classical cell cycle arrest of CDK4/6i, namely the senescence-associated secretory phenotype that it induced. Senescence-associated secretory phenotype has a dual role in cancer treatment.^[62]^

## 4. Discussion and future outlook

Currently, CDK4/6i is the universal first-line clinical standard for HR+/HER2− MBC in combination with endocrine therapy. Palbociclib in combination with aromatase inhibitors have been approved by the Chinese FDA for first-line treatment in postmenopausal HR+/HER2− patients with locally advanced or metastatic BC. Moreover, malignant tumors are prone to natural or acquired resistance to CDK4/6is, which affects the therapeutic effect and its clinical application. However, the problems arising from resistance to CDK4/6i are new. Because the cell cycle control is a classic sign of cancer, for hormone drug resistance in patients with metastatic BC, has proven to be an effective target, CDK4/6i mediated cell cycle arrest and aging need to complete the cell cycle pathway. The nonspecific cell cycle mechanism has also been found to mediate CDK4/6i resistance, the most typical of which is the PI3K/Akt/mTOR pathway. The inhibitors targeting this target have achieved promising results in preclinical studies on combination therapy with CDK4/6i. For example, CDK4/6 targeted therapies not only exert immune regulatory effects on tumor cells, but also produce complex immune regulatory networks on tumor microenvironment immune cells, and the role of CDK4/6i and apoptosis and senescence of BC cells in drug resistance remains to be studied.

Most of researchers have high expectations for CDK4/6i/ET/PI3Ki triple therapy, but it is difficult to solve the problem of CDK4/6i resistance of patients through a single reverse resistance strategy. A combination of biomarkers or gene expression levels is needed to accurately target therapy. In addition, it is still controversial whether to reverse resistance after CDK4/6i resistance, or to carry out preventive reversal resistance strategy in the first line of advanced BC, which involves the differences among patients with primary resistance and acquired resistance. Moreover, for any kind of multi-drug combination therapy, the adverse reaction caused by multi-drug combination is absolutely a problem that cannot be ignored, and it becomes a challenge to take safety and efficacy into consideration. Therefore, further researches on mechanisms and clinical validation were needed to develop successful treatment strategies to overcome CDK4/6i resistance.

## Author contributions

**Conceptualization:** Wu Jiayi, Wang Wei, Shao Xiying, Wang Xiaojia.

**Data curation:** Wu Jiayi, Lin Guang.

**Writing – original draft:** Wu Jiayi, Wang Wei.

**Writing – review & editing:** Wu Jiayi, Wang Wei.
